# Reassessment of Neuronal Tau Distribution in Adult Human Brain and Implications for Tau Pathobiology

**DOI:** 10.1186/s40478-022-01394-9

**Published:** 2022-06-28

**Authors:** Giavanna Paterno, Brach M. Bell, Kimberly-Marie M. Gorion, Stefan Prokop, Benoit I. Giasson

**Affiliations:** 1 Department of Neuroscience, College of Medicine, University of Florida, Gainesville, FL 32610 USA; 2 Center for Translational Research in Neurodegenerative Disease, College of Medicine, University of Florida, BMS J483/CTRND, 1275 Center Drive, Gainesville, FL 32610 USA; 3 College of Medicine, McKnight Brain Institute, University of Florida, Gainesville, FL 32610 USA; 4 Department of Pathology, University of Florida, Gainesville, FL 32610 USA; 5 Fixel Institute for Neurological Diseases, University of Florida, Gainesville, FL 32610 USA

**Keywords:** Brain, Human, Neuronal, Distribution, Tau, Alzheimer’s disease, Tauopathy

## Abstract

Tau is a predominantly neuronal, soluble and natively unfolded protein that can bind and stabilize microtubules in the central nervous system. Tau has been extensively studied over several decades, especially in the context of neurodegenerative diseases where it can aberrantly aggregate to form a spectrum of pathological inclusions. The presence of tau inclusions in the form of neurofibrillary tangles, neuropil threads and dystrophic neurites within senile plaques are essential and defining features of Alzheimer’s disease. The current dogma favors the notion that tau is predominantly an axonal protein, and that in Alzheimer’s disease there is a redistribution of tau towards the neuronal soma that is associated with the formation of pathological inclusions such as neurofibrillary tangles and neuropil threads. Using novel as well as previously established highly specific tau antibodies, we demonstrate that contrary to this overwhelmingly accepted fact, as asserted in numerous articles and reviews, in adult human brain, tau is more abundant in cortical gray matter that is enriched in neuronal soma and dendrites compared to white matter that is predominantly rich in neuronal axons. Additionally, in Alzheimer’s disease tau pathology is significantly more abundant in the brain cortical gray matter of affected brain regions compared to the adjacent white matter regions. These findings have important implications for the biological function of tau as well as the mechanisms involved in the progressive spread of tau associated with the insidious nature of Alzheimer’s disease.

**Supplementary Information:**

The online version contains supplementary material available at 10.1186/s40478-022-01394-9.

## Introduction

**Fig. 1:**
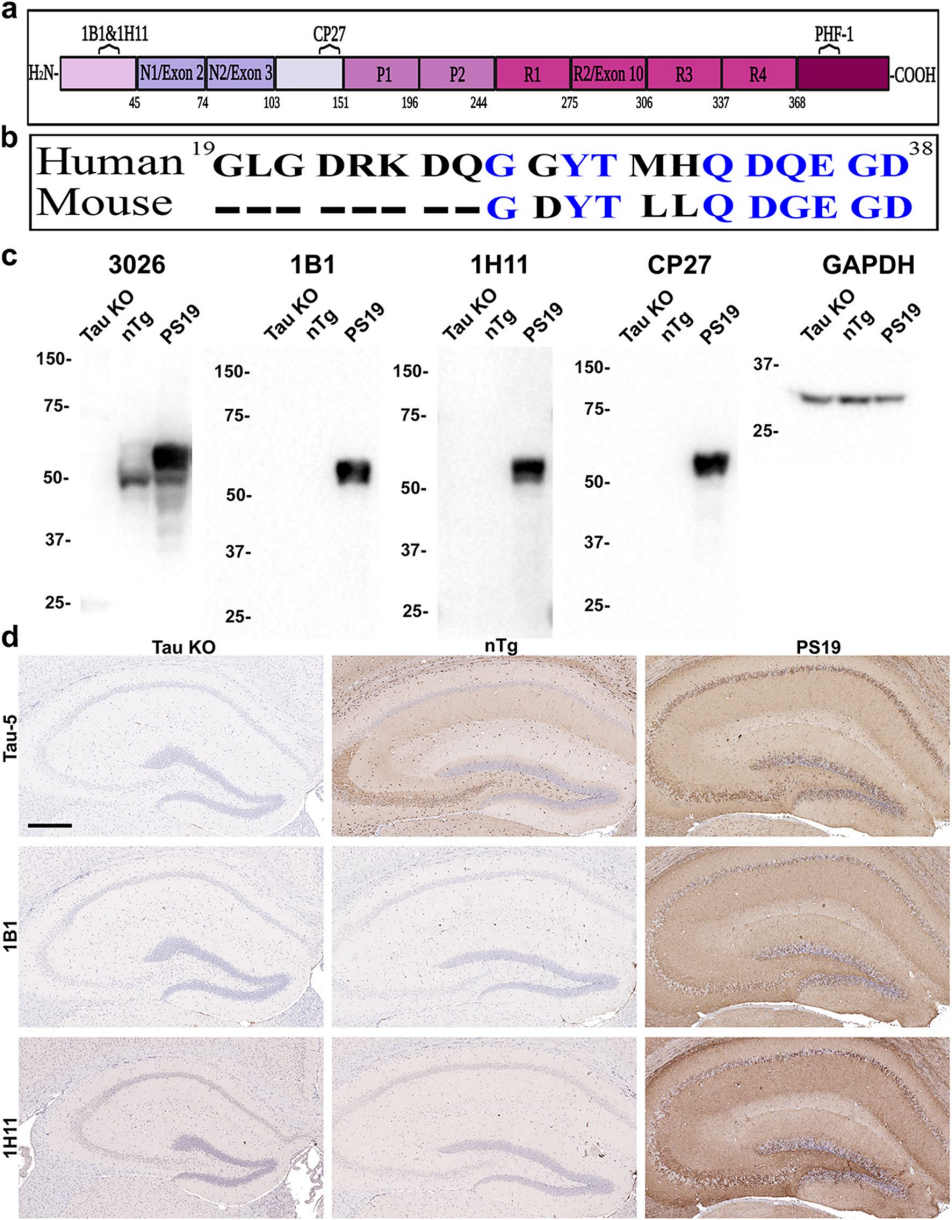
Characterization of the specificity of the new tau antibodies 1B1 and 1H11. **a** Schematic of full-length human tau (2N4R) with the location of the antibody epitopes used in the study. Created with BioRender.com. **b** Amino acid sequence of human and mouse tau corresponding to residues 19 to 38 in human tau. Non-homologous amino acids between species are indicated in black lettering. Dashes indicate residues that are not present in mouse tau. **c** 10 µg of total brain lysate from tau KO, nTg, and PS19 mice were resolved by SDS-PAGE as indicated above each lane and used for immunoblotting as described in Materials and Methods. Blots were probed with antibodies 3026 (mouse and human tau), 1B1 (human tau), 1H11 (human tau), CP27 (human tau) and anti-GAPDH as a loading control. The mobility of protein markers with their molecular masses are indicated on the left side of each immunoblot. **d** IHC was performed as described in Materials and Methods. Brains of tau KO, nTg, and PS19 mice were stained with the total tau antibody Tau-5, and the two novel monoclonal total human tau antibodies, 1B1 and 1H11. Sections were counterstained with hematoxylin. Scale bar indicates 300 $\mu \mathrm{m}$

Tau is a microtubule (MT) associated protein that is predominately expressed in neurons in the human central nervous system (CNS) [74]. The physiological functions of tau include promoting tubulin polymerization and MT stability, which are mediated through the MT binding repeat domain of tau and the upstream adjacent proline rich domains [12, 17, 40]. The expression of tau is developmentally regulated and in human adult brain tau presents as 6 isoforms through alternative splicing of exons 2, 3, and 10 which lead to tau proteins having 0, 1, or 2 N terminal inserts and 3 or 4 MT binding repeats (see Fig. 1) [21, 33]. Under non-pathological conditions, tau exists predominantly as a soluble and natively unfolded protein [6, 44] which lacks secondary structure and discontinuously associates and regulates MTs [45]. Under pathological conditions, including Alzheimer’s disease (AD), tau forms insoluble aggregates such as paired helical filaments and straight filaments which form the components of neurofibrillary tangles (NFT) [12, 20, 21, 32, 34, 41, 44, 47, 52, 56, 74, 80]. The pathological hallmarks of AD include amyloid β (Aβ) deposits known as senile plaques and pathological tau inclusions that present as NFT, neuropil threads, and dystrophic neurites within senile plaques [24, 28, 51].

The presence and accumulation of insoluble tau aggregates is also found in other neurodegenerative diseases collectively termed tauopathies [12, 30, 53, 74]. The mechanism(s) through which tau aggregation is prompted and causes neurodegeneration in AD and other neurodegenerative diseases is still unknown, but a leading hypothesis is that tau pathology propagates through a prion-like mechanism and spreads transcellularly through connected brain regions [4, 30, 62].

The neuronal distribution of tau was initially described in rat and bovine brain and was found to be predominantly localized in axons [7]. The current dogma posits that under pathological conditions, where tau is aggregated and highly phosphorylated, it is aberrantly re-localized to the somatodendritic compartment of the neuron [2, 71, 74, 85]. While our understanding of tau pathology has been furthered by the availability of tau antibodies generated using synthetic peptides and AD brain lysate, a limitation of these antibodies is that many cross-react with other microtubule associated proteins (MAPs) due to the homology in the MT binding region [8, 57, 73]. Furthermore, binding of many tau antibodies depends on the phosphorylation state of the targeted epitopes and thus staining with these antibodies does not reveal the distribution of all tau species [2, 61, 64, 69]. In the current study, we generated and characterized two novel monoclonal tau antibodies raised against a phosphorylation independent epitope in the N terminal domain which do not cross react with other MAPs. Histologically and biochemically, it is demonstrated that in the brains of healthy adult controls, tau is more abundant in the cerebral cortical gray matter where neuronal soma and dendrites are located rather than the cerebral cortical white matter that is highly enriched in axons. A similar distribution was observed in the cortical subregions of AD brains where pathological tau aggregation also is predominantly observed in the gray matter compared to white matter. These findings have important implications for the normal biological function of tau in the adult human brain as well as the underlying mechanisms resulting in pathological tau aggregation including a critical reshaping of the prion-type spreading model.

## Materials and methods

### Mice

All animal experimental procedures were performed in accordance to University of Florida Institutional Animal Care and Use Committee regulatory policies following approval. BALB/c mice, tau knockout (KO) [22] and PS19 tau transgenic mice [84] were obtained from Jackson Laboratories (Bar Harbor, ME). For immunohistochemistry (IHC), mice were euthanized with CO_2_, perfused with a heparin/PBS solution, fixed in formalin and embedded in paraffin before staining.

### Generation of new N-terminal Tau monoclonal antibodies

Monoclonal antibodies were generated as previously described [66]. In brief, a synthetic peptide (CGLGDRKDQGGYTMHQDQEGD) corresponding to amino acids 19–38 of human tau was synthesized and purified by GenScript USA, Inc. (Piscataway, NJ). This peptide contains an added cysteine residue at the amino-terminus that allows for conjugation to Imject maleimide-activated mariculture keyhole limpet hemocyanin (mcKLH; Termo Scientifc, Waltham, MA). The peptides conjugated to mcKLH were used to immunize female BALB/c mice (Jackson Laboratory, Bar Harbor, ME).

Hybridoma clones were screened by enzyme-linked immunosorbent assay (ELISA) with 96-well ELISA plates (Thermo Fisher Scientifc, Waltham, MA) coated with 1 µg/ml of peptide in PBS using the unconjugated peptide used from immunization. Wells were washed with PBS and blocked with PBS/5% fetal bovine serum (FBS). Media from hybridomas were added to plates and incubated at room temperature. Plates were washed with PBS and were then incubated with horseradish peroxidase-conjugated anti-mouse antibody (Jackson Immuno Research Labs, West Grove, PA) in 5% FBS/PBS for an hour. Following washes with PBS, 3,3′,5,5′-tetramethylbenzidine (TMB substrate, Thermo Fisher Scientifc, Waltham, MA) was added to each well until a color change was observed. The reactions were stopped by adding 0.5 M HCl and the optical density was measured at 450 nm with a plate reader. Positive clones were further tested by ELISA using recombinant tau protein as well as synthetic peptide (AGTYGLGDRKDQGG) corresponding to amino acid residues 15–28 in human tau. The isotypes of antibodies 1B1 and 1H11 were determined to be IgG_1_ using a mouse monoclonal isotyping kit (Millipore Sigma, Burlington, MA).

### Preparation of total protein lysate from mouse brain tissue

Mice were euthanized with CO_2_ and the brains from PS19 tau transgenic mice, tau KO mice, and non-transgenic (nTg) mice were harvested. Mouse brain were disrupted by sonication in 2% SDS/50 mM Tris, pH 7.5 and the lysates were heated to 95 $^\circ$C for 10 min. Protein concentrations were determined using BCA assay using bovine serum albumin as the standard (Thermo Fisher Scientific, Waltham, MA). SDS sample buffer was added to the lysates and heated to 95 $^\circ$C for 10 min and stored at − 80 °C.

### Immunohistochemistry

Tissue sections from paraffin-embedded blocks were rehydrated in xylene and a series of descending alcohols (100–70%) and washed in water. Heat-induced epitope retrieval (HIER) was performed in a steam bath for 60 min for human tissue or 30 min for mouse tissue in a citrate solution (Target Retrieval Solution Citrate pH 6; Agilent, Santa Clara, CA). Next, slides were washed in water and endogenous peroxidases were quenched by incubating sections in 1.5% hydrogen peroxide in PBS (Invitrogen, Waltham, MA). Sections were then rinsed in water and incubated in 0.1 M Tris and blocked in 2% FBS/0.1 M Tris. Primary antibodies were diluted in blocking solution or applied undiluted and were added onto tissue sections at 4 °C overnight. Slides were washed in 0.1 M Tris and incubated with biotinylated anti-mouse secondary antibody (1:3000) and immPRESS HRP horse anti-mouse polymer detection (1:5) (Vector Laboratories; Burlingame, CA) diluted in blocking solution for 1 h at room temperature. Slides were subsequently washed with 0.1 M Tris and incubated with an avidin–biotin complex (Vectastain ABC Elite kit; Vector Laboratories, Burlingame, CA) for 1 h at room temperature. Immunocomplexes were detected with the chromogen 3,3′-diaminobenzidine (DAB kit; KPL, Gaithersburg, MD). Sections were counterstained with Mayer’s hematoxylin (Sigma Aldrich, St. Louis, MO), washed in water, dehydrated in a series of ascending alcohols (70–100%) and xylenes, and coverslipped using Cytoseal (Thermo Scientific, Waltham, MA).

### Sequential brain fractionation for isolation of soluble and insoluble Tau

Frozen human brain was obtained from the University of Florida Neuromedicine Human Brain and Tissue Bank (Table 1). Gray and white matter from the temporal and occipital brain cortices were carefully dissected, weighed and disrupted by sonication in 6 mL of high-salt (HS) buffer (50 mM Tris–HCl, pH 7.5, 0.75 M NaCl, 2 mM EDTA) containing a protease inhibitor cocktail (1 mM phenylmethylsulfonyl fluoride and 1 $\upmu$g/ml each of pepstatin, leupeptin, *N*-tosyl-l-phenylalanyl chloromethyl ketone, *N*-tosyl-lysine chloromethyl ketone, and soybean trypsin inhibitor) per gram of tissue. The lysate was centrifuged at 100,000 × g at 4 $^\circ$C for 30 min. The supernatants were kept as the HS soluble fractions and the pellets were homogenized in 4 mL of HS buffer with 1% Triton buffer per gram of tissue, and centrifuged at 100,000× *g* at 4 $^\circ$C for 30 min. These fractions were kept as the HS/1% Triton-soluble fractions and the pellet was homogenized in 6 mL HS buffer with 1% Triton and 1 M sucrose, and centrifuged at 100,000 × g at 4 $^\circ$C for 30 min to float the myelin. This fraction was discarded, and the pellet was homogenized in 2 mL of HS buffer with 1% Sarkosyl per gram of tissue, incubated at 37 $^\circ$C for 30 min and centrifuged at 100,000 × g at 4 $^\circ$C for 30 min. This fraction was kept as the HS/Sarkosyl soluble fractions and the pellets were sonicated in 1 mL SDS/urea (2% SDS, 4 M urea, 25 mM Tris–HCl pH 7.6) per gram of tissue, centrifuged at 100,000 × g at 25 $^\circ$C for 30 min. This fraction was collected as the SDS/urea soluble fraction. SDS was added to the HS, Triton/HS, and Sarkosyl/HS fractions, for a final concentration of 2% SDS. These were heated at 95 °C for 10 min and all fractions were kept frozen in -80 °C till further use. Protein concentrations were determined using bicinchoninic acid (BCA) assay using bovine serum albumin as the standard (ThermoFisher Scientific, Waltham, MA). SDS sample buffer was added to the HS soluble/2% SDS, Triton/HS/2% SDS, and Sarkosyl/HS/2%SDS fractions that were incubated for 10 min at 95 $^\circ$C. SDS sample buffer was added to the SDS/urea soluble samples that were incubated for 10 min at 45 $^\circ$C.

**Table 1:** Information on the patient tissue used in the studies

Cases	Neurological diagnosis	Primary neuropathological diagnosis	Secondary neuropathological diagnosis	Thal	Braak	CERAD	APOE	Sex	Age
CTL-1		CVD	AD low	1	II	none	3/3	F	90
CTL-2		PART, Braak I	CAA widespread, moderate	0	I	none	3/3	M	71
CTL-3		PART, Braak II		0	II	none	3/3	F	72
AD-1	AD	AD high	CAA widespread, mild	4	V	frequent	3/4	M	77
AD-2	AD	AD high	CAA widespread, severe	5	V	frequent	3/4	F	72
AD-3	AD	AD high	CAA widespread, moderate	5	VI	frequent	3/4	M	63

Listed are the clinical and pathological diagnoses, sex, age, APOE genotype status, and Thal, Braak, and CERAD ratings. *AD* Alzheimer’s disease, *CTL* control, *CVD* cerebrovascular disease, *PART* primary age-related tauopathy, *CAA* cerebral amyloid angiopathy, *APOE* apolipoprotein E

### Immunoblotting

5 µg of protein extracts from human tissue and 10 µg of protein extracts from mouse tissue were loaded on 8% SDS–polyacrylamide gels, separated by PAGE, and electrophoretically transferred onto 0.45 µm nitrocellulose membranes. The membranes were blocked in 5% non-fat milk in Tris-buffered saline (TBS) for 1 h at room temperature and incubated in primary antibody diluted in milk overnight at 4 $^\circ$C. Membranes were washed in TBS and incubated in anti-mouse or anti-rabbit secondary antibodies conjugated to horseradish peroxidase (Jackson ImmunoResearch, West Grove, PA). Following washing in TBS, the membranes were imaged by chemiluminescence using Western Lighting Plus ECL reagents (PerkinElmer Life Sciences, Waltham, MA) and a GeneGnome XRQ imaged system (Syngene, Frederick, MD).

### Additional antibodies

Mouse monoclonal antibodies, CP27 and PHF-1 were generous gifts from the late Dr. Peter Davies [26, 60]. Total Tau 3026 rabbit polyclonal antibody was previously described [68, 81]. Mouse monoclonal antibody Tau-5 was purchased from Invitrogen (Waltham, MA). Mouse monoclonal anti-NFL antibody NR4 and mouse monoclonal anti-NFM antibody NN18 were obtained from Sigma (St. Louis, MO). A mouse monoclonal antibody specific for glyceraldehyde-3-phosphate dehydrogenase (GAPDH, clone GA1R) was from Fisher Scientific.

### Expression and purification of recombinant human tau isoforms

Human tau isoforms were cloned into the pRK172 bacterial expression vector using *NdeI* and *EcoRI* restriction sites as previously described [27, 31, 75]. Recombinant tau isoforms were expressed in *Escherichia coli* BL21 (New England Biolabs, Ipswich, MA) and purified. In brief, expression was induced by isopropyl-β-D-1-thiogalactopyranoside (IPTG) and high-salt 100 °C heat stable bacterial protein lysates were purified by MonoS cation exchange chromatography.

### Analysis of tissue staining and immunoblots

All slides were digitally scanned using an Aperio ScanScope CS instrument (40× magnification; Aperio Technologies Inc., Vista, CA), and images of antibody staining were captured using the ImageScope software (40× magnification; Aperio Technologies Inc). Immunoblots and IHC images were individually white leveled. Brightness and contrast corrections were applied to each IHC figure using Adobe Photoshop (Adobe Systems, San Jose,CA,USA). Raw files are available upon request.

## Results

### Generation and characterization of novel tau monoclonal antibodies with epitopes in the amino-terminal region

The amino-terminal region of tau is the least studied region of this molecule [12, 55] and there are relatively few available antibodies that target this region. Mice were immunized with a peptide corresponding to residues 19–38 in human tau, numbered according to the 2N/4R human tau isoform, that is upstream of the N1 and N2 alternatively spliced protein segments (Fig. 1a). Two monoclonal antibodies 1B1 and 1H11 were identified by ELISA screen with the original peptide used for immunization as well as recombinant tau proteins. These antibodies recognize all 6 major tau isoforms expressed in the human CNS (Additional file 1: Fig. S1). ELISAs using a peptide spanning residues 15–28 in human tau reveal that these antibodies did not recognize this region of tau (data not shown) indicating that their epitopes include residues 29–38, that has 2 amino acid difference between human and mouse tau (Fig. 1b). To further characterize the specificity of the new antibodies, immunoblotting and IHC were performed using brain tissue from tau KO [22], non-transgenic (nTg), and PS19 tau transgenic mice [84] (Fig. 1c, d). PS19 mice express 1N/4R human tau with the P301S mutation. By immunoblotting, tau that is predominantly expressed as the 0N/4R isoform in adult mouse brain [58] and human 1N/4R transgenic tau in PS19 mice were detected with total tau antibody 3026 (Fig. 1c). Antibodies 1B1 and 1H1 only reacted with human tau and similar to antibody CP27 that is also human tau specific but with an epitope at residues 130–150 in the middle region of tau [26]. IHC analyses also demonstrated that antibodies 1B1 and 1H11 only specifically stained human tau as compared to antibody Tau-5 that reacts with both mouse and human tau [65] (Fig. 1d).

### Immunohistochemical analysis with novel antibodies reveal that tau is more abundant in cortical gray matter relative to white matter in human brain

**Fig. 2:**
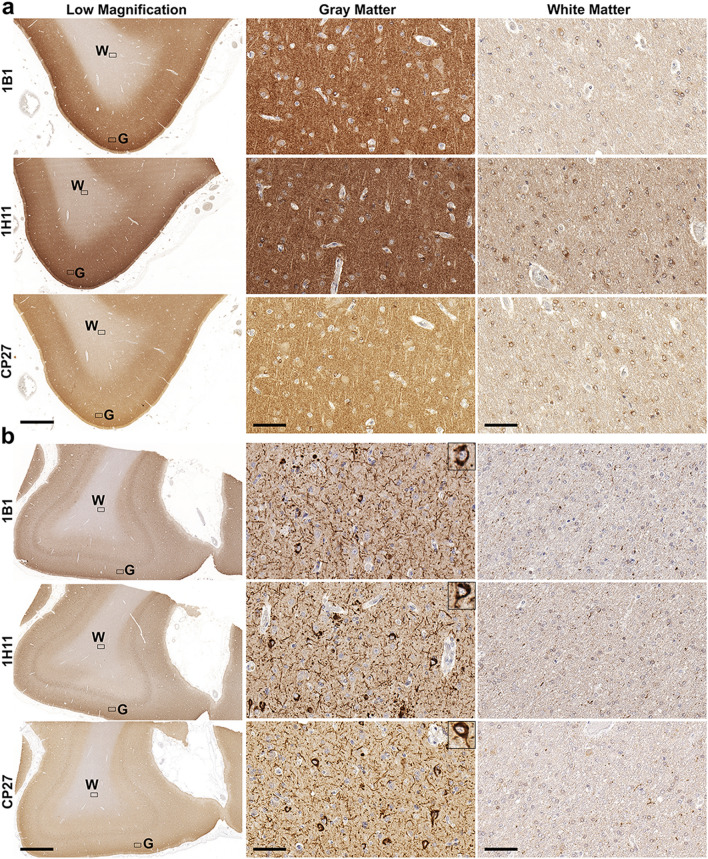
Immunohistochemistry of the frontal cortex of a control individual and an AD individual with phospho-independent tau antibodies. IHC was performed as described in Materials and Methods. A representative control case **(a)** and a representative AD case **(b)** were stained with total human tau antibodies 1B1, 1H11, and CP27. Low magnification and high magnification images of gray matter and white matter were taken as indicated. “G” indicates gray matter region. “W” indicates white matter region. Scale bar for low magnification images is 2 mm. Scale bar for high magnification images is 60 µm; and insets 30 µm. Sections were counterstained with hematoxylin

**Fig. 3:**
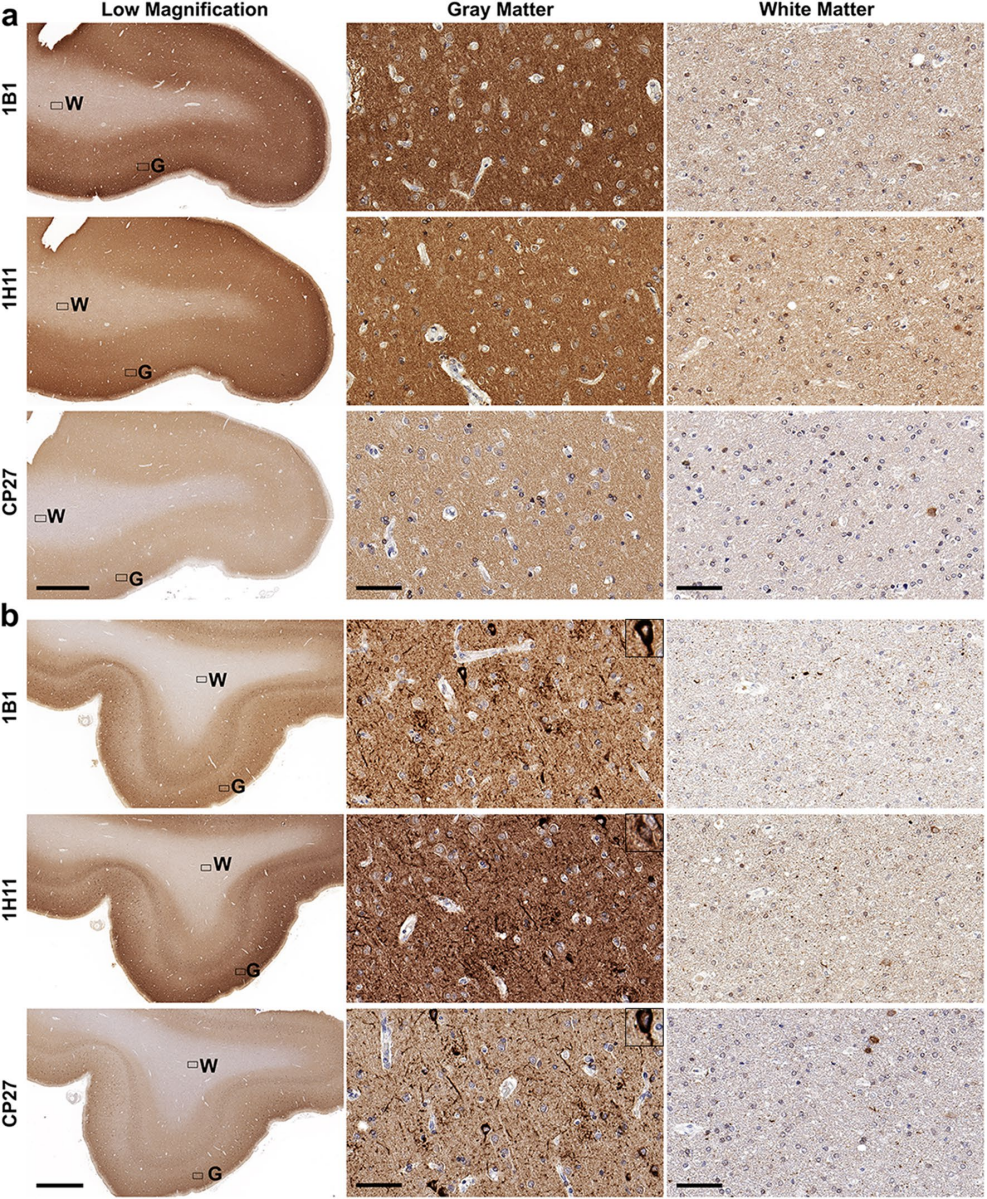
Immunohistochemistry of the temporal cortex of a control individual and an AD individual with phospho-independent tau antibodies. IHC was performed as described in Materials and Methods. A representative control case **(a)** and a representative AD case **(b)** were stained with total human tau antibodies 1B1, 1H11, and CP27. Low magnification and high magnification images of gray matter and white matter were taken as indicated. “G” indicates gray matter region. “W” indicates white matter region. Scale bar for low magnification images is 2 mm. Scale bar for high magnification images is 60 µm; and insets 30 µm. Sections were counterstained with hematoxylin

Staining of control cases with new tau antibodies 1B1 and 1H11 revealed predominant staining of cortical gray matter regions (Figs. 2, 3, Additional file 1: S2 and S3). In fact, the cortical gray and white matter were easily distinguished in control tissue from all four regions by staining with these new tau antibodies, which was surprising given the current dogma that tau is predominately an axonal protein (Figs. 2, 3, Additional file 1: S2 and S3). Staining of advanced stages (Braak V/VI) AD brain tissue (Table 1) with new tau antibodies 1B1 and 1H11 labelled NFT, neuropil threads, and dystrophic neurites within senile plaques (Figs. 2, 3, Additional file 1: S2 and S3). While staining in the white matter region revealed sparse tau immunoreactive glial inclusions and processes, in AD brain tissue these antibodies revealed much more intense staining of the gray matter neuropil regions relative to the white matter regions in the frontal cortex (Fig. 2), temporal cortex (Fig. 3), parietal cortex (Additional file 1: Fig. S2), and occipital cortex (Additional file 1: Fig. S3). A similar staining pattern was observed with another human tau antibody, CP27, and this clear difference in tau staining pattern was also observed among brain regions of control and AD individuals (Figs. 2, 3, Additional file 1: S2 and S3). In the cortical gray matter of control cases, neuronal cell bodies, dendrites, and unmyelinated axons were stained in all four regions with the new N-terminal tau antibodies and CP27.

Most antibodies typically used to detect tau pathological inclusions in human brain rely on conformation dependent epitopes or tau phospho-epitopes, as tau can be phosphorylated at many sites that become hyperphosphorylated in disease conditions [77, 83]. Therefore, many antibodies specific for phosphorylated tau have been used to detect pathological inclusions, but these cannot be used to inform us on the normal distribution of tau. Staining of AD brain tissue with one of these antibodies PHF-1, specific for tau phosphorylated at Ser396 and Ser404, revealed NFT, neuropil threads and dystrophic neurites with more robust labeling of the gray matter compared to white matter areas (Additional file 1: Fig. S4). But this difference in staining in AD tissue is confounded by the pathological accumulation of tau in the gray matter as well as tau’s phosphorylation state. In brain tissue from control individuals, a difference in PHF-1 staining intensity between gray and white matter could be observed but it was less obvious than with the phosphorylation independent tau antibodies. In addition, to ensure that this distinct staining pattern was not due to the IHC methodology, human brain tissue was also stained for another neuronal cytoskeletal protein, the neurofilament medium molecular mass subunit (NFM) showing equivalent reactivity in all gray and white brain cortical regions (Additional file 1: Fig. S5).

### Biochemical fractionation and immunoblotting analyses reveal that tau is more abundantly present in the gray cortical cerebral tissue than the adjacent white matter tissue

**Fig. 4:**
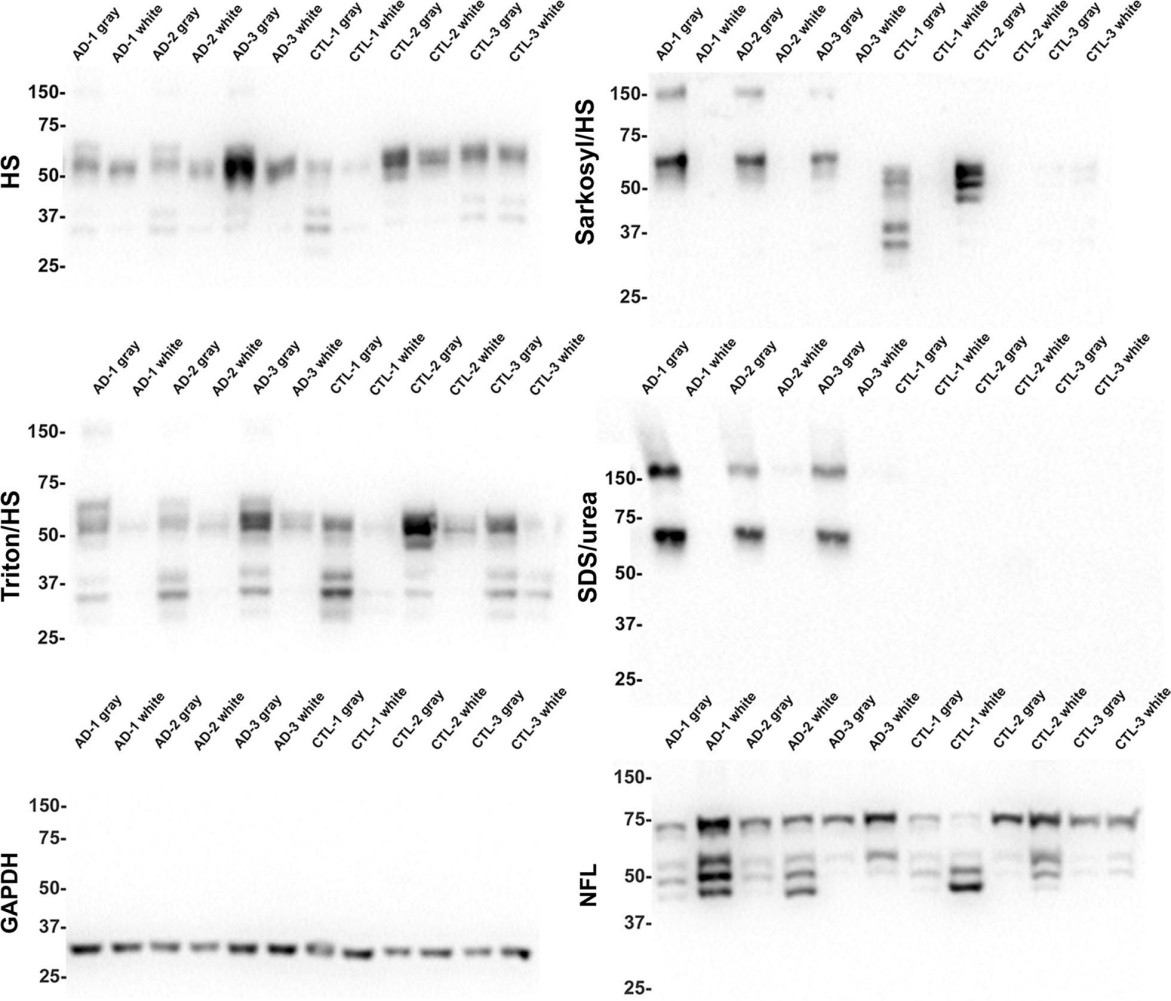
Comparative biochemical fraction analyses of gray and white matter from the temporal cortex of AD and control cases with anti-tau antibody 1B1. Biochemical fractionation of AD and CTL cerebral temporal cortex was performed as described in Materials and Methods. Equal amount of protein (5 $\upmu$g) from the high salt (HS) soluble, Triton/HS soluble, Sarkosyl/HS soluble, and Sarkosyl-insoluble SDS/urea soluble fractions were separated by SDS-PAGE and analyzed by immunoblotting with tau antibody, 1B1. The HS fractions were also probed for GAPDH and the SDS/urea fractions were probed for NFL. The mobility of protein markers with their molecular masses are indicated on the left side of each immunoblot

To further investigate the distribution of tau in adult human brain that was revealed by the IHC studies, gray and white matter form the temporal cortex of control and AD brains was carefully dissected and biochemically fractionated (Additional file 1: Fig. S6) followed by immunoblotting analyses. Cortical gray matter is enriched in neuronal cell bodies and dendrites, while the subcortical white matter is enriched in myelinated axons. Immunoblotting with the new tau antibodies 1B1 (Fig. 4) and 1H11 (Additional file 1: Fig. S7) reveal that tau, which migrates as multiple bands around 55 kDa, was predominately found in the HS and Triton/HS fractions and was more abundant within the gray matter cerebral brain cortical tissue compared to the white matter cerebral brain cortical tissue in control brain tissue in the temporal brain region. Several low molecular weight bands between 25 and 37 kDa were observed in the gray and white matter, but these bands were more pronounced in the gray matter. These low molecular weight bands are predicted to be proteolytically cleaved tau products. A similar relative abundance in the gray versus white matter tissue was also observed in AD temporal cortical brain tissue. In AD temporal cortical brain tissue tau was also found in the Sarkosyl/HS-soluble and SDS/urea fractions, predominantly in the gray matter. An additional band at ~ 150 kDa was observed in the Sarkosyl/HS soluble and SDS/urea fractions from AD cases.

**Fig. 5:**
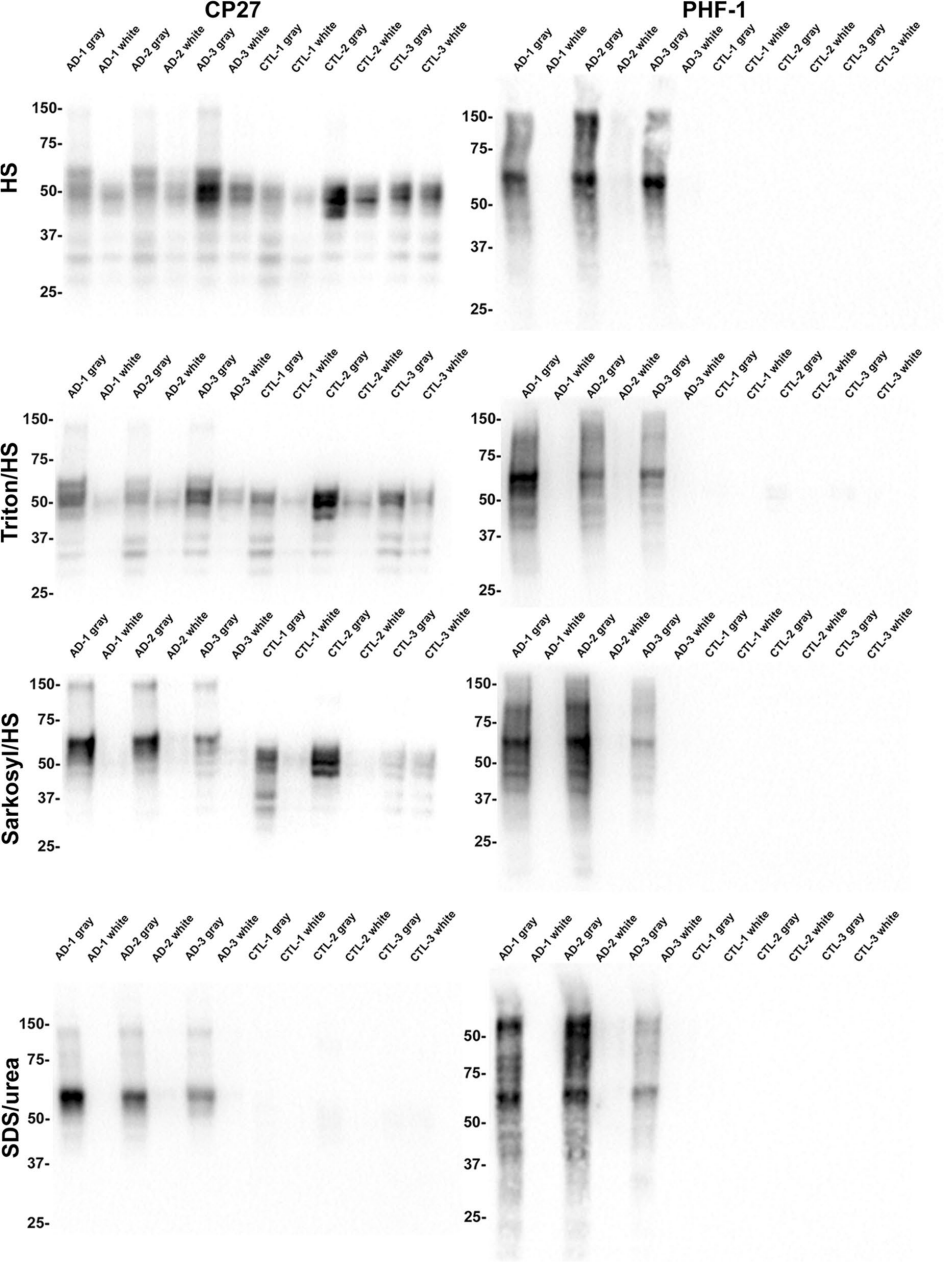
Comparative biochemical fraction analyses of gray and white matter from the temporal cortex of AD and control cases with tau antibodies CP27 and PHF-1. Biochemical fractionation of AD and CTL cerebral temporal cortex was performed as described in Materials and Methods. Equal amount of protein (5 $\upmu$g) from the high salt (HS) soluble, Triton/HS soluble, Sarkosyl/HS soluble, and Sarkosyl-insoluble SDS/urea soluble fractions were separated by SDS-PAGE and analyzed by immunoblotting with antibodies CP27 or PHF-1. The mobility of protein markers with their molecular masses are indicated on the left side of each immunoblot

These studies were expanded to assess if these tau distribution patterns were also observed in the occipital cortex (Additional file 1: Fig. S8 and S9). Consistent results were obtained where tau was more abundant in the gray matter than the white matter and accumulated as biochemically Triton X-100 insoluble aggregates in AD tissue. To further investigate this distribution of tau in the temporal cortex, the biochemical fractions were also analyzed with another phospho-independent antibody, CP27, where similar results were obtained analyzing biochemical fractions from the temporal (Fig. 5) and occipital cortex (Additional file 1: Fig. S10). As tau in AD brain is hyperphosphorylated, the biochemical fractions were also probed with phosphorylation specific antibody, PHF-1. Hyperphosphorylated tau was predominantly observed in the gray matter of AD individuals within both regions, as indicated by a smear in the gray matter lanes with one case also showing some pathological tau in the white matter in the occipital cortex (Fig. 5, Additional file 1: Fig. S10).

## Discussion

Tau was first identified in 1975 as a MAP which colocalizes with MTs and promotes tubulin polymerization reaction [76]. Tau was subsequently further purified and confirmed to have a pivotal role in MT formation [18, 79]. Following the advent of tau monoclonal antibodies raised from immunization with human brain homogenate, it was initially observed by IHC that tau is localized in axons [7, 50, 63]. Many others groups have conducted studies to try to elucidate the distribution of tau using both IHC and biochemical methodology. Consistent with our findings, one group performed IHC staining which found “normal” tau to localize in the neuropil in several brain regions of adult individuals, with limited staining in axons [72]. Other groups performed biochemistry on gray and white matter from adult human frontal cortex from AD and control individuals [46] and bovine brain [1] and found tau to be highly localized in the gray matter. While these studies cast doubt on the current dogma of tau’s axonal localization, they were limited by their use of antibodies which are now known to either cross-reacts with other MAPs, are dependent on tau’s phosphorylation state, or do not react with all tau isoforms. Despite these contradictory results on the neurobiology of tau protein, it still widely accepted as a protein highly enriched in axons with limited involvement in the soma of neurons [7, 25, 35, 39, 42, 50, 63]; and thus tau’s distribution is thought to change under pathological conditions in which tau is re-localized to accumulate towards the soma of neurons [2, 3, 23, 36, 37, 48, 49, 54, 70, 71, 74, 85, 86]. However, the interpretations of many earlier studies were limited due to the fact that many available tau antibodies are dependent on the phosphorylation state of tau or can cross-react to other non-tau proteins such as other MAPs that share sequence homology to tau.

In our study, we evaluated the distribution of tau protein under normal and pathological conditions in the adult human brain using monoclonal antibodies that are not phosphorylation dependent and with epitopes outside of the MT binding region. We used two novel total tau antibodies raised against a phosphorylation independent epitope shared by all 6 tau isoforms in the N-terminus and another phosphorylation independent antibody CP27 with the epitope in the middle region of tau. We demonstrated that these antibodies are highly specific for tau.

IHC was used to assess the distribution of tau in control and AD brain cortical tissue from four distinct cortical regions (temporal, frontal, parietal, and occipital regions) that are differentially affected during the progression of AD as described by Braak staging [10]. We observed strong IHC staining for tau in the neuropil within the gray matter region of control brains in all four regions studied. The staining in the gray matter region was significantly more intense than in the adjacent white matter. In AD tissue, staining for tau in the gray matter was also more intense than in the white matter. Additional immunoreactivity for NFT, neuropil threads, and dystrophic neurites were observed in the cortical gray matter.

The enrichment of tau in the cortical gray matter tissue compared to white matter was confirmed by biochemical fractionation followed by immunoblotting. Sequentially fractionated tau was found to be more abundant in the gray matter which was observed using our new phosphorylation-independent tau antibodies. Soluble tau bands were located at molecular weights of ~ 55 kD as well as 25–37 kD. Tau is known to be cleaved by proteases such as caspases and cathepsins present in neurons which could potentially accounts for these lower molecular weight species [82]. As expected, these studies revealed that Sarkosyl-insoluble tau is present in AD brain tissue but not in controls. However, in AD brains, HS and HS/Triton soluble tau was also present in a more highly phosphorylated state compared to controls as shown with antibody PHF-1. It is not clear if this finding reflects smaller tau aggregates that did not sediment during fractionation or hyperphosphorylated monomeric tau. Collectively, our findings have significant implication for the current dogma about normal tau biology and its pathobiology. Our findings demonstrate that under normal conditions in the human adult brain tau is more abundant in the cortical gray matter region, enriched in neuronal soma and dendrites than the region (white matter) that is predominantly comprised of axons.

Tau has been most vastly studied for its functions as a neuronal MAP where is can regulate MT dynamics and MT interactions with other proteins. Within the somatodendritic compartment of neurons tau can facilitate cellular trafficking as a MAP [5, 13], but it can have many other functions as it known to interact with other neuronal proteins such as actin, other MAPs, scaffolding proteins, protein kinases and phosphatases that are present throughout neurons consistent with tau’s physiological distribution observed herein [39].

Our findings challenge the prevailing notion that tau is mostly an axonal protein under physiological conditions which it is redistributed towards the soma and dendrites in AD brains. Furthermore, many studies have indicated that tau aggregation can exhibit prion-like behavior in which tau competent seeds are able to induce a conformational change in endogenous tau that propels its neuroanatomical pathological spread [4, 11, 14–16, 19, 29, 30, 38, 43, 59, 67, 78] reflecting the stage-wise progression of tau pathology described by Braak staging [9, 10]. Using phospho-specific tau antibodies such as PHF-1 or AT8, as well as silver staining, Braak and others demonstrated that tau pathology progressively spreads in the gray matter, typically starting in the entorhinal cortex region of the hippocampal formation [9, 10]. Using highly specific phospho-independent tau antibodies by IHC as well as biochemical fractionation/immunoblotting analysis, we further confirm that pathological tau is predominantly present in the cortical gray matter. The higher abundance of tau in the gray matter likely contributes to its preferential aggregation and spread in these brain regions compared to white matter. However, if prion-like progression plays a major role in the progression of tau pathology it is still puzzling that tau pathology is sparse in the axons of the white matter. In primary neuronal cell culture studies it has been shown that tau aggregates can travel down axons and even seed the formation of tau inclusions in post-synaptic neurons [30, 38]. Perhaps in human adult brain, tau within the white matter axonal compartment is not available for recruitment by tau prion-type seeds as it is mainly sequestered onto MTs or the mechanisms involved in intercellular transmission of seeds occurs preferentially between cell bodies and dendrites. In AD, A$\upbeta$ brain deposition also occurs predominantly in the gray matter region compared to the white matter so perhaps A$\upbeta$ plays a critical role in dictating the directionality of pathological tau spread. Maybe the accumulation of A$\upbeta$ deposits enhance the neuronal activities involved in the secretion/uptake of tau seeds or promote the cellular apparatus that can directly result in intercellular exchange such as nanotubes [30, 38]. These hypotheses will have to be investigated in future studies, but the current findings presented here have important implication for basic tau biology and AD pathogenesis as well as therapeutic targeting.

## Conclusions

The data presented here underscore that the current dominant notions on the normal neuronal distribution and function of tau in the adult human brain need significant adjustments. Our data indicate that in adult human brain cortical neurons tau has a more prevailing abundance within the somatodendritic compartments compared to axons. This physiological distribution of tau that mirrors the neuroanatomical deposition of tau inclusion pathology in AD brains also has important implications for the current models on the progressive spread of tau pathology in neurodegenerative diseases.

## Supplementary Information

**Additional file 1:** Additional data on the characterization of novel tau antibodies and the distribution of tau in human brains by immunohistochemistry and biochemical analyses.

## Abbreviations

Aβ: Amyloid β
ABC: Avidin–biotin complex
AD: Alzheimer’s disease
CTL: Control
CNS: Central nervous system
DAB: 3,3′-Diaminobenzidine
FBS: Fetal bovine serum
IHC: Immunohistochemistry
KO: Knockout
MAP: Microtubule associated protein
MT: Microtubule
NFT: Neurofibrillary tangles
nTg: Non-transgenic
TBS: Tris-buffered saline

## Declarations

### Availability of data and materials

The datasets used and analyzed during the current study are available from the corresponding author on reasonable request.
