# Supplementary material for: Reassessment of Neuronal Tau Distribution in Adult Human Brain and Implications for Tau Pathobiology
Source: Acta Neuropathol Commun. 2022 Jun 28;10:94. doi: 10.1186/s40478-022-01394-9 (PMC9237980; doi:10.1186/s40478-022-01394-9)
Supplement: Supplementary file 1 — Additional file 1: Additional data on the characterization of novel tau antibodies and the distribution of tau in human brains by immunohistochemistry and biochemical analyses. [file 40478_2022_1394_MOESM1_ESM.pdf]

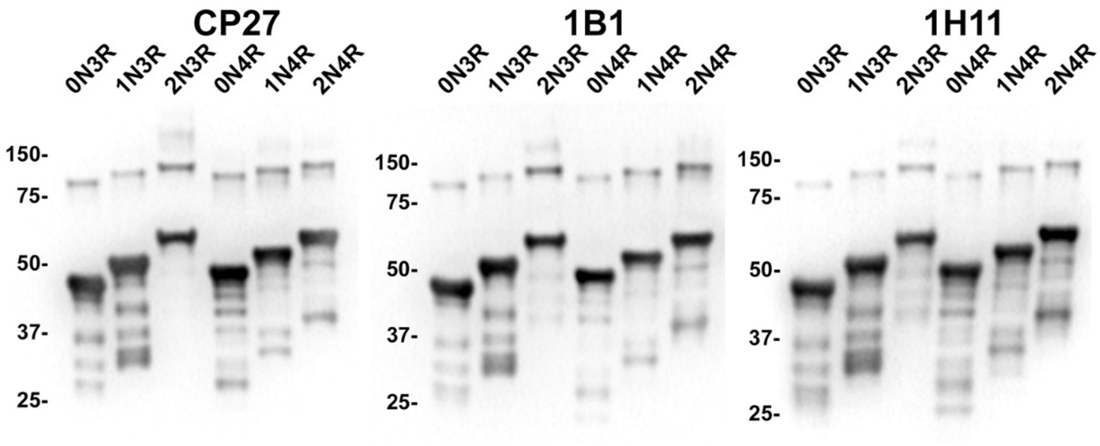

Fig S1. Immunoblots with tau antibodies using recombinant human tau proteins. The recombinant forms of the six CNS human tau isoforms were produced as described in Materials and Methods. 200 ng of each tau isoform was loaded on SDS-polyacrylamide gels as indicated above each lane and electophoretically transferred to nitrocellulose membranes. Blots were probed with total human tau antibodies CP27, 1B1, and 1H11. The mobility of protein markers with their molecular masses are indicated on the left side of each immunoblot.

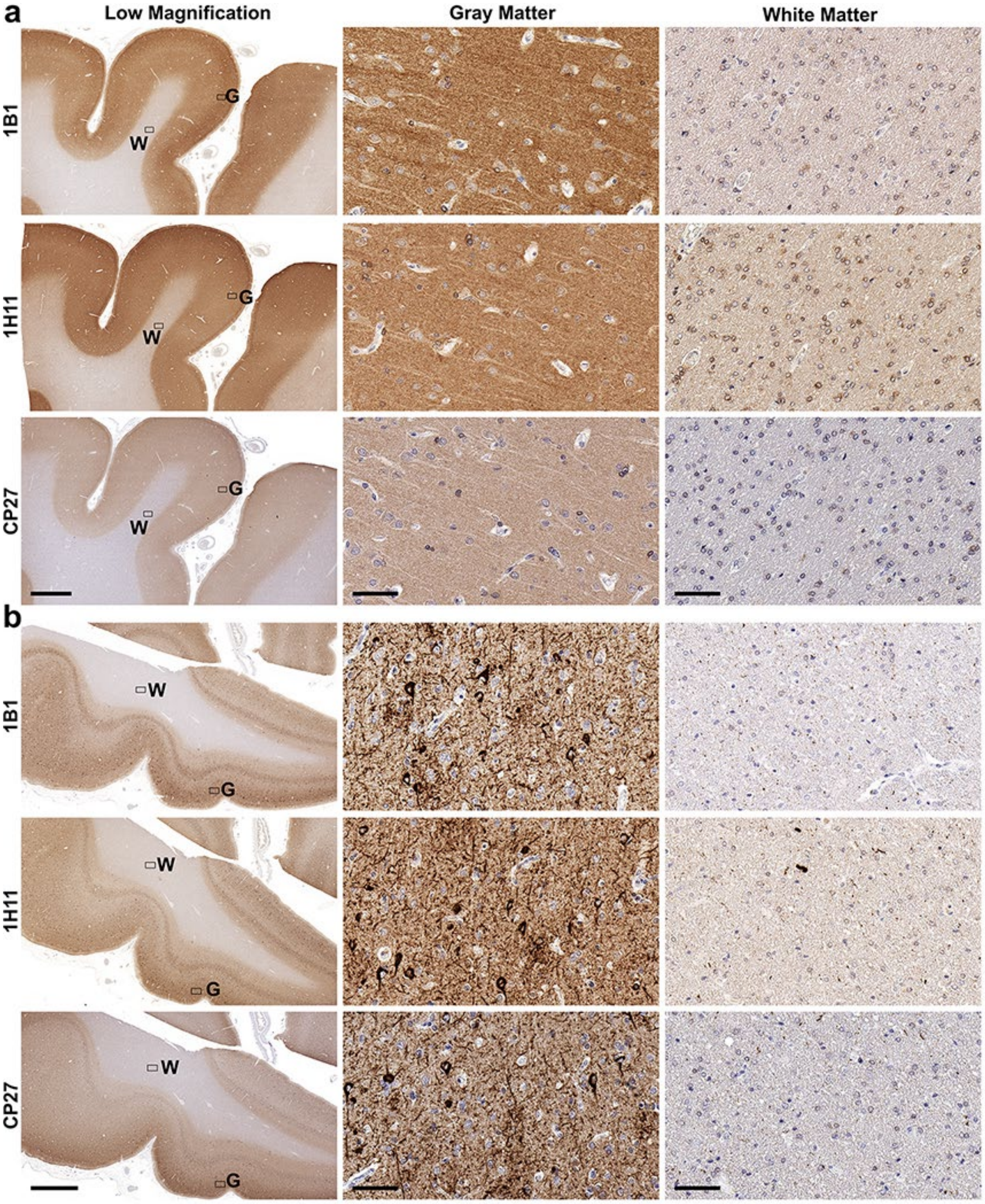

Fig S2. Immunohistochemistry of the parietal cortex of a control individual and an AD individual with phospho-independent tau antibodies. IHC was performed as described in Materials and Methods. A representative control case **(a)** and a representative AD case **(b)** were stained with total human tau antibodies 1B1, 1H11, and CP27. Low magnification and high magnification images of gray matter and white matter were taken as indicated. "G" indicates gray matter region. "W" indicates white matter region. Scale bar for low magnification images is 2 mm. Scale bar for high magnification images is 60 µm. Sections were counterstained with hematoxylin.

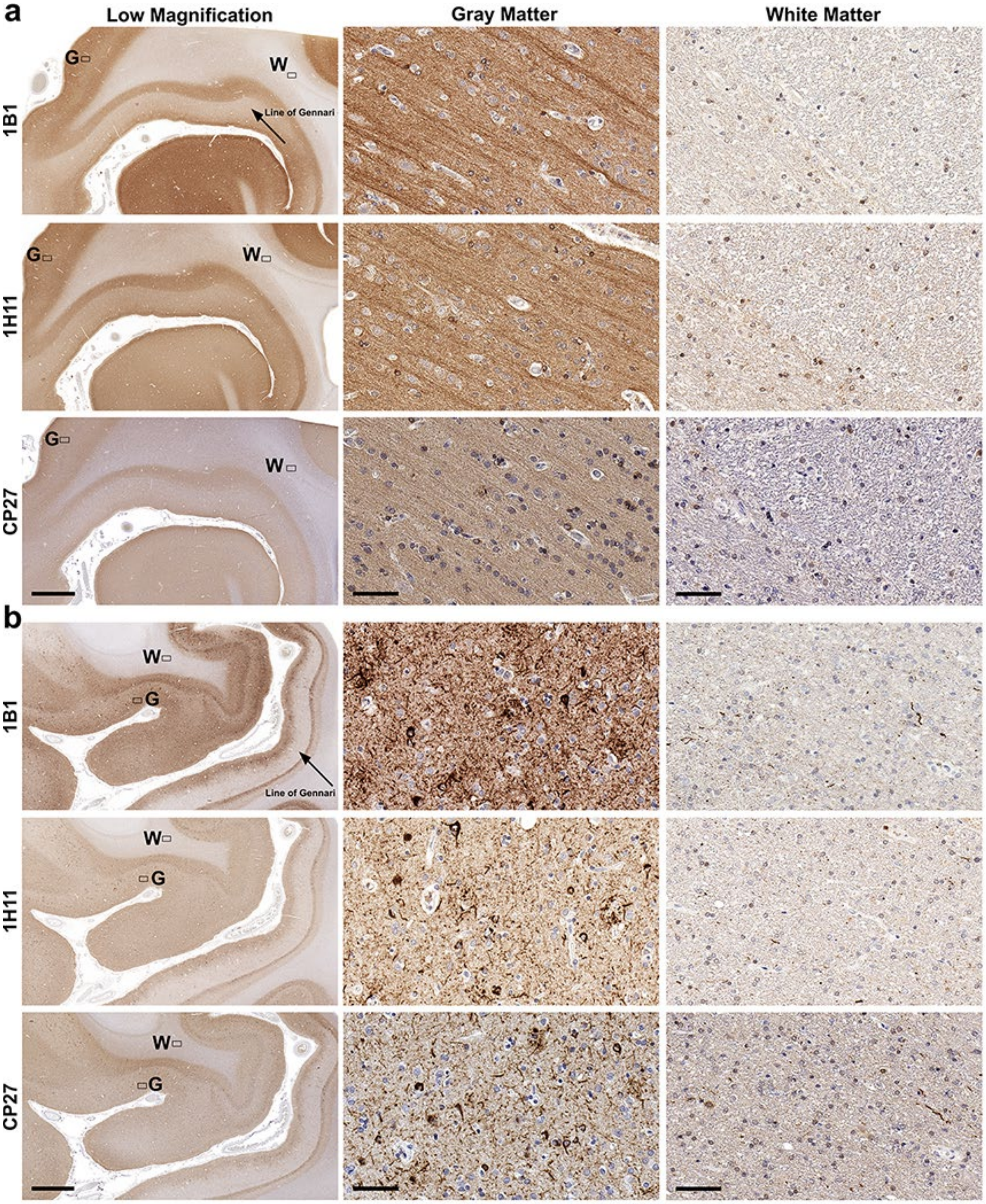

Fig S3. Immunohistochemistry of the occipital cortex of a control individual and an AD individual case with phospho-independent tau antibodies. IHC was performed as described in Materials and Methods. A representative control case **(a)** and a representative AD case **(b)** were stained with total human tau antibodies CP27, 1B1, and 1H11. Low magnification and high magnification images of gray matter and white matter were taken as indicated. "G" indicates gray matter region. "W" indicates white matter region. Scale bar for low magnification images is 2 mm. Scale bar for high magnification images is 60 μm. Sections were counterstained with hematoxylin.

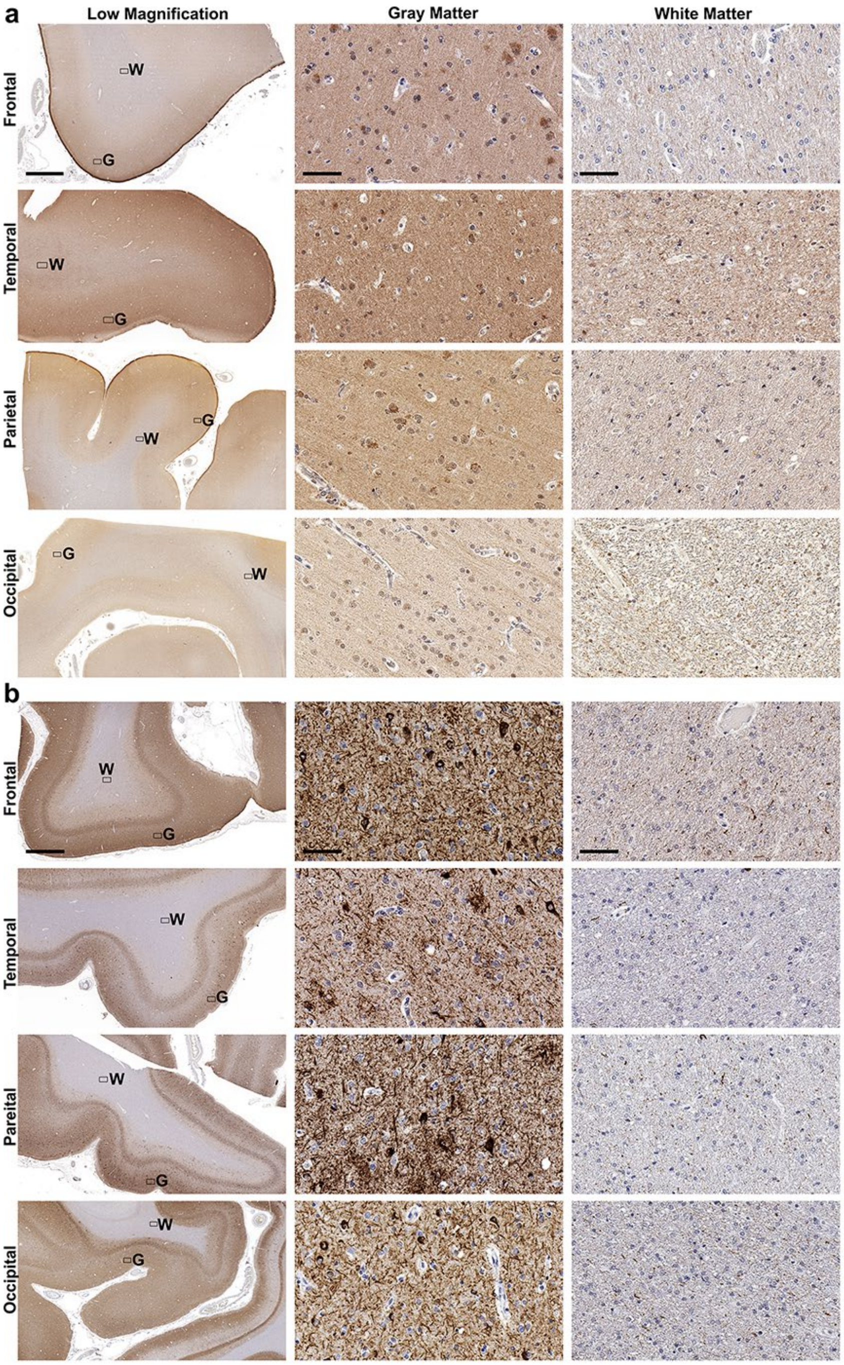

Fig S4. Immunohistochemistry of the frontal, temporal, parietal, and occipital cerebral cortex of a control individual and an AD individual with tau phospho-dependent antibody PHF-1. IHC was performed as described in Materials and Methods. A representative control case **(a)** and a representative AD case **(b)** were stained with the phosphorylation specific (pS396/pS404) tau antibody, PHF-1. Low magnification and high magnification images of gray matter and white matter were taken as indicated. “G” indicates gray matter. “W” indicates white matter. Scale bar for low magnification images is 2 mm. Scale bar for high magnification images is 60 µm. Sections were counterstained with hematoxylin.

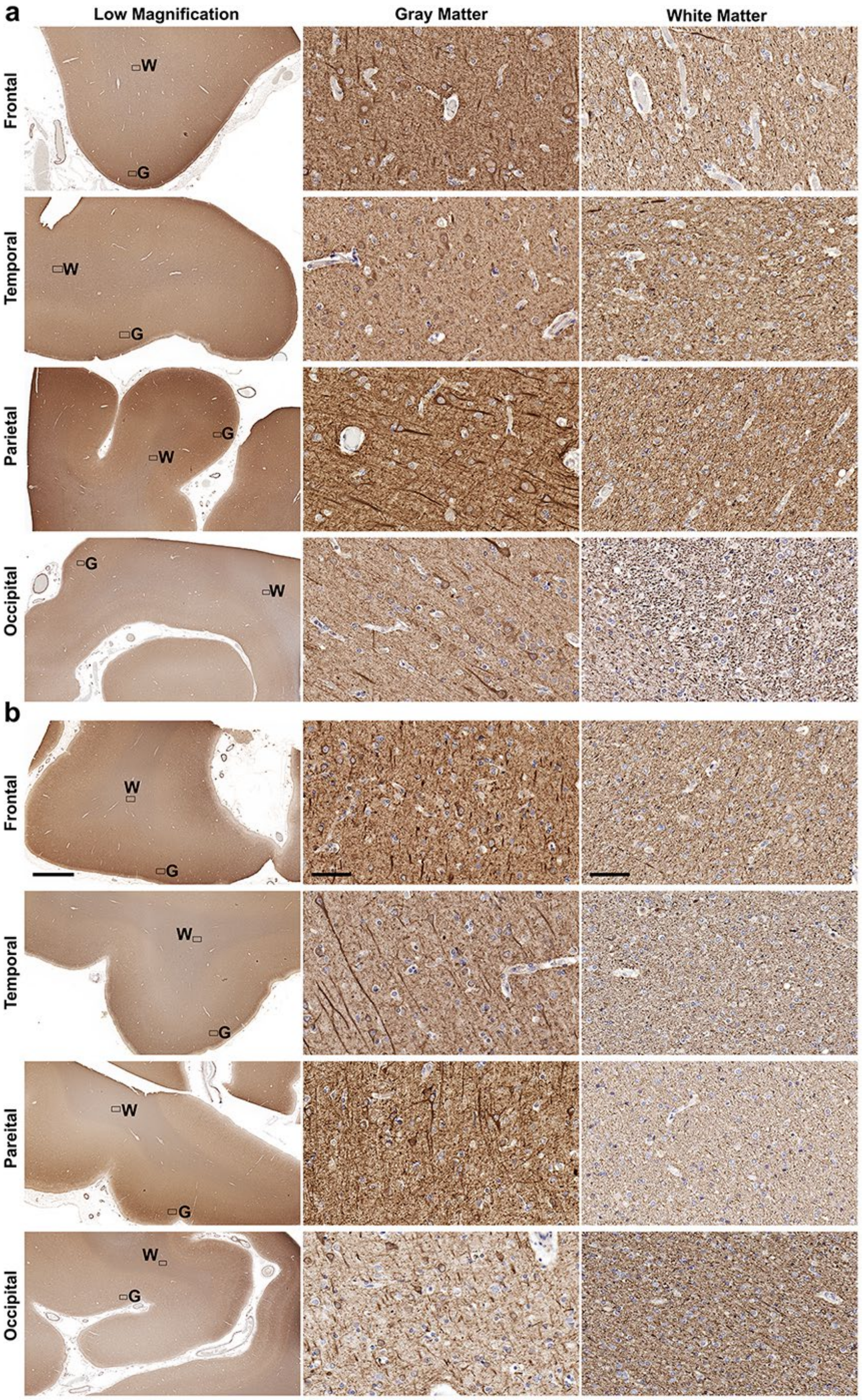

Fig S5. Immunohistochemistry of the frontal, temporal, parietal, and occipital cerebral cortex of a control individual and an AD individual with anti-NFM antibody NN18.
IHC was performed as described in Materials and Methods. A representative control case **(a)** and representative AD case **(b)** were stained with anti-NFM specific antibody, NN18. Low magnification and high magnification images of gray matter and white matter were taken as indicated. "G" indicates gray matter region. "W" indicates white matter region. Scale bar for low magnification images is 2 mm. Scale bar for high magnification images is 60 µm. Sections were counterstained with hematoxylin.

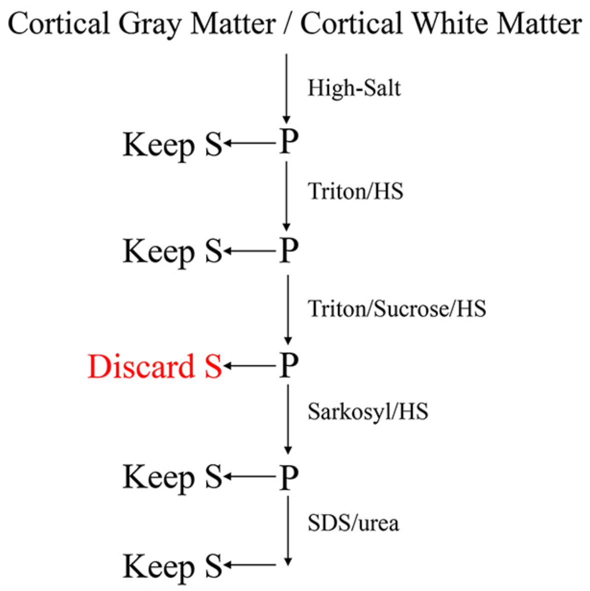

Fig S6. Schematic of human brain fractionation process. Sequential tau fractionation of human cortical gray and white matter. "S" indicates supernatant. "P" indicates pellet.

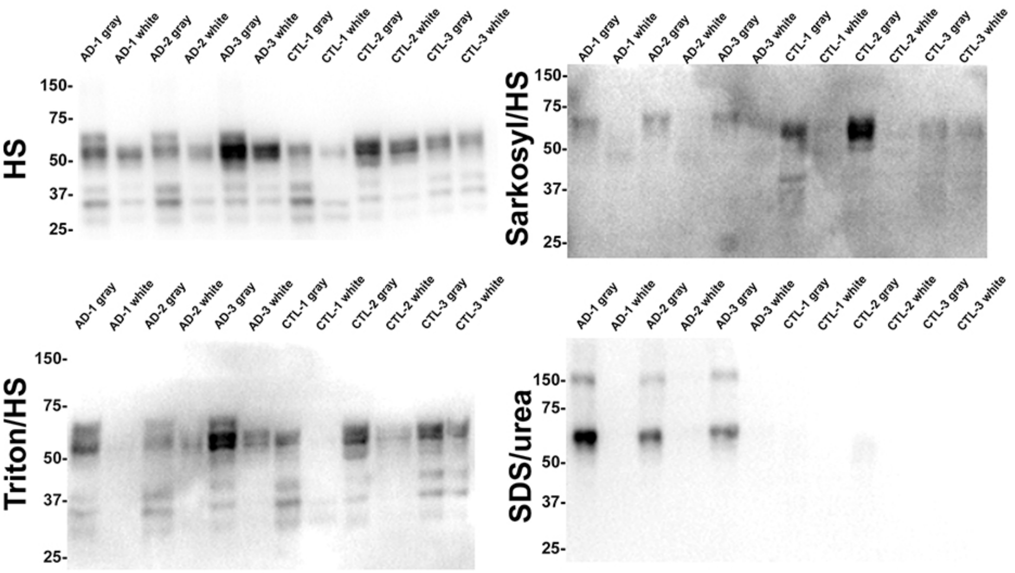

Fig S7. Comparative biochemical fraction analyses of gray and white matter from the cerebral temporal cortex of AD and control cases with anti-tau antibody 1H11. Biochemical fractionation of AD and CTL cerebral temporal cortex was performed as described in Materials and Methods. Equal amount of protein (5 μg) from the high salt (HS) soluble, Triton/HS soluble, Sarkosyl/HS soluble, and Sarkosyl-insoluble SDS/urea soluble fractions were separated by SDS-PAGE and analyzed by immunoblotting with tau antibody, 1H11. The mobility of protein markers with their molecular masses are indicated on the left side of each immunoblot.

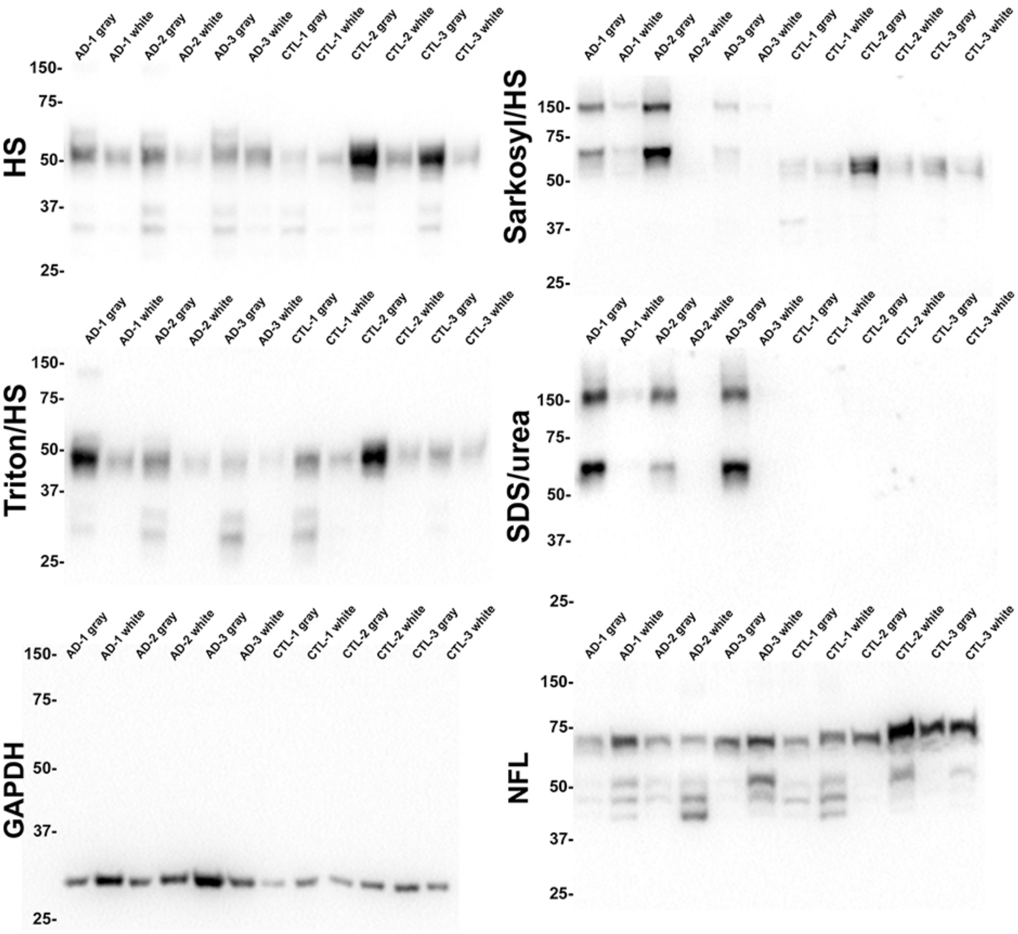

Fig S8. Comparative biochemical fraction analyses of gray and white matter from the occipital cortex of AD and control cases with anti-tau antibody 1B1. Biochemical fractionation of AD and CTL cerebral temporal cortex was performed as described in Materials and Methods. Equal amount of protein (5 μg) from the high salt (HS) soluble, Triton/HS soluble, Sarkosyl/HS soluble, and Sarkosyl-insoluble SDS/urea soluble fractions were separated by SDS-PAGE and analyzed by immunoblotting with tau antibody, 1B1. The HS fractions were also probed for GAPDH and the SDS/urea fractions were probed for NFL. The mobility of protein markers with their molecular masses are indicated on the left side of each immunoblot.

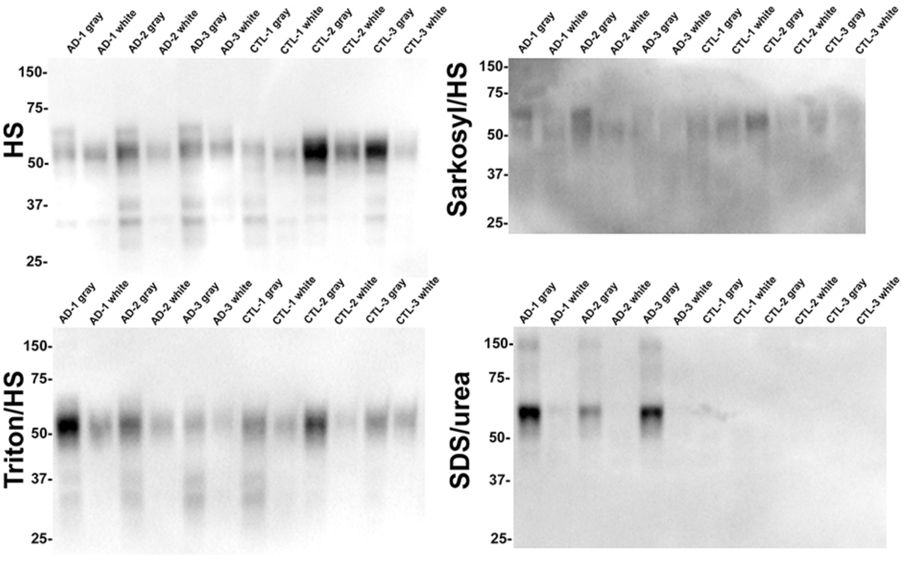

Fig S9. Comparative biochemical fraction analyses of gray and white matter from the cerebral occipital cortex of AD and control cases with anti-tau antibody 1H11. Biochemical fractionation of AD and CTL cerebral temporal cortex was performed as described in Materials and Methods. Equal amount of protein (5 μg) from the high salt (HS) soluble, Triton/HS soluble, Sarkosyl/HS soluble, and Sarkosyl-insoluble SDS/urea soluble fractions were separated by SDS-PAGE and analyzed by immunoblotting with tau antibody, 1H11. The mobility of protein markers with their molecular masses are is indicated on the left side of each immunoblot.

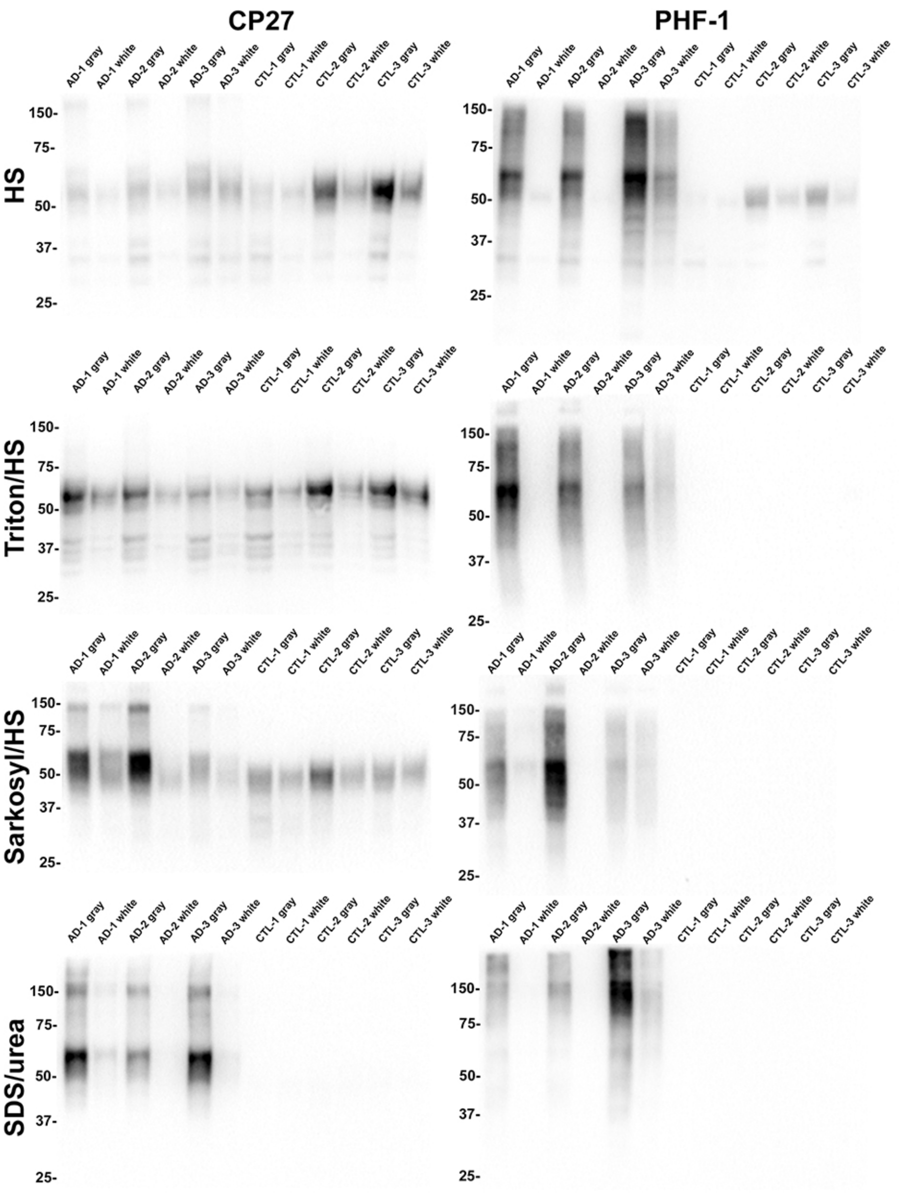

Fig S10. Comparative biochemical fraction analyses of gray and white matter from the occipital cortex of Alzheimer's disease and control cases with antibodies CP27 and PHF-1. Biochemical fractionation of AD and CTL cerebral occipital cortex was performed as described in Materials and Methods. Equal amount of protein (5 μg) from the high salt (HS) soluble, Triton/HS soluble, Sarkosyl/HS soluble, and Sarkosyl-insoluble SDS/urea soluble fractions were separated by SDS-PAGE and analyzed by immunoblotting with antibodies CP27 or PHF-1. The mobility of protein markers with their molecular masses are indicated on the left side of each immunoblot.
